# (*E*)-4-[(5-Methyl-2-fur­yl)methyl­ene­amino]benzene­sulfonic acid

**DOI:** 10.1107/S1600536808026275

**Published:** 2008-08-20

**Authors:** Jianlan Suo

**Affiliations:** a Department of Chemistry, Baoji University of Arts and Science, Baoji, Shaanxi 721007, People’s Republic of China

## Abstract

The title compound, C_12_H_11_NO_4_S, is a Schiff base derived from the condensation reaction of equimolar quanti­ties of sulfamide and furfural. The mol­ecule has a *trans* configuration with respect to the imine C=N double bond. The N atom is involved in an inter­molecular O—H—N hydrogen bond.

## Related literature

For related literature, see: Abd El Rehim *et al.* (2001 ▶); Hariharan & Urbach (1969 ▶); Koning & Canti­lena (1994 ▶); Tarafder *et al.* (2002 ▶).
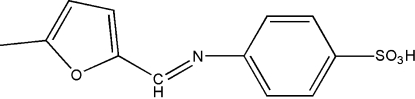


## Experimental

### 

#### Crystal data

C_12_H_11_NO_4_S
*M* *_r_* = 265.28
Monoclinic, 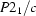

*a* = 13.9761 (11) Å
*b* = 11.9820 (15) Å
*c* = 7.3266 (10) Å
β = 95.8010 (10)°
*V* = 1220.6 (2) Å^3^
*Z* = 4
Mo *K*α radiation
μ = 0.27 mm^−1^
*T* = 298 (2) K
0.23 × 0.20 × 0.15 mm

#### Data collection

Bruker SMART CCD area-detector diffractometer
Absorption correction: multi-scan (*SADABS*; Sheldrick, 1996 ▶) *T*_min_ = 0.940, *T*_max_ = 0.961
6042 measured reflections
2156 independent reflections
1581 reflections with *I* > 2σ(*I*)
*R*_int_ = 0.028

#### Refinement

*R*[*F* ^2^ > 2σ(*F* ^2^)] = 0.044
*wR*(*F* ^2^) = 0.128
*S* = 1.05
2156 reflections
165 parameters
H-atom parameters constrained
Δρ_max_ = 0.43 e Å^−3^
Δρ_min_ = −0.44 e Å^−3^

### 

Data collection: *SMART* (Bruker, 2000 ▶); cell refinement: *SAINT* (Bruker, 2000 ▶); data reduction: *SAINT*; program(s) used to solve structure: *SHELXS97* (Sheldrick, 2008 ▶); program(s) used to refine structure: *SHELXL97* (Sheldrick, 2008 ▶); molecular graphics: *SHELXTL* (Sheldrick, 2008 ▶); software used to prepare material for publication: *SHELXTL* (Sheldrick, 2008 ▶).

## Supplementary Material

Crystal structure: contains datablocks I, global. DOI: 10.1107/S1600536808026275/bx2168sup1.cif

Structure factors: contains datablocks I. DOI: 10.1107/S1600536808026275/bx2168Isup2.hkl

Additional supplementary materials:  crystallographic information; 3D view; checkCIF report

## Figures and Tables

**Table 1:** Hydrogen-bond geometry (Å, °)

*D*—H⋯*A*	*D*—H	H⋯*A*	*D*⋯*A*	*D*—H⋯*A*
O1—H1⋯N1^i^	0.82	2.21	3.025 (3)	176

Symmetry code: (i) 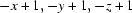.

**Fig. 1.**
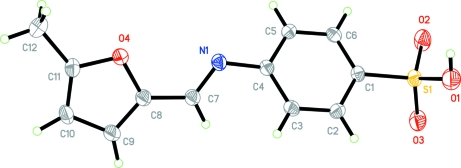
The structure of the title compound in 30% probability ellipsoids. H atoms are shown as spheres of arbitrary radii.

**Fig. 2.**
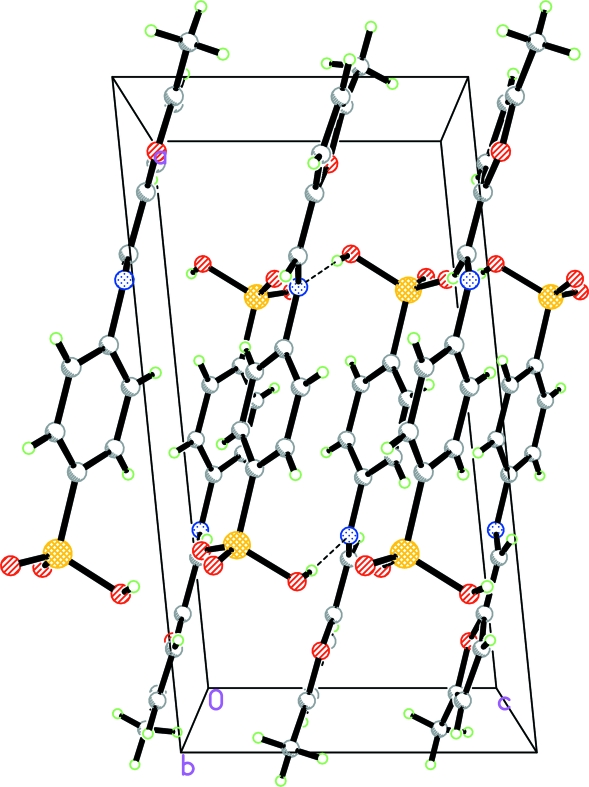
The molecular packing of (I) viewed along the b axis. The dotted lines represent hydrogen bonds. [Symmetry code: (A)-x + 1, y, -z + 1]

## supplementary crystallographic information

### Comment

In the past decade, Schiff base compounds have been of great interest. These compounds play an important role in the development of coordination chemistry. Some of the complexes derived from Schiff bases have been found to the complexes with pharmacological and antitumor properties (Abd El Rehim *et al.*, 2001; Koning & Cantilena, 1994; Tarafder *et al.*, 2002). As an extension of the work on the structural characterization of Schiff base compounds, the crystal structure of the title compound, (I), is reported here.

The title compound (I) is an sulfamide derivative. The molecular structure is shown in Fig.1. All bond lengths and bond angles are in the normal ranges and comparable to those observed in a similar sulfamide Schiff base (Hariharan & Urbach 1969). The dihedral angle between the benzene ring and the furfural system is 31.9 (6)°. The torsion angles of N1—C4—C5—C6 and N1—C7—C8—C9 are 178.8 (2) ° and -174.2 (3) °, respectively. The molecular structure adopts a *trans* configuration about the C7 ═ N1 bond. Table 1 shows hydrogen-bond geometry and a packing diagram is shown in Fig.2.

### Experimental

Sulfamide (0.1 mmol, 17.2 mg) and furfural(0.1 mmol, 9.6 mg) were dissolved in methanol (10 ml). The mixture was stirred for 30 min at room temperature to give a clear brown solution. After allowing the resulting solution to stand in air for 7 d, brown flake-shaped crystals of (I) were formed on slow evaporation of the solvent. The crystals were collected, washed with methanol and dried in a vacuum desiccator using anhydrous CaCl_2_ (yield 54%). Analysis found: C 54.28%, H 4.15% calculated for C_12_H_11_NO_4_S: C 54.34%, H 4.15%.

### Refinement

All H atoms were placed in geometrically idealized positions and constrained to ride on their parent atoms with C—H distances in the range 0.93–0.96Å and *U*_iso_(H) = 1.2*U*_eq_ or 1.5*U*_eq_(C/O)

### Crystal data

**Table:**

C_12_H_11_NO_4_S	*F*_000_ = 552
*M**_r_* = 265.28	*D*_x_ = 1.444 Mg m^−^^3^
Monoclinic, *P*2_1_/*c*	Mo *K*α radiation λ = 0.71073 Å
*a* = 13.9761 (11) Å	Cell parameters from 1942 reflections
*b* = 11.9820 (15) Å	θ = 2.9–26.0º
*c* = 7.3266 (10) Å	µ = 0.27 mm^−^^1^
β = 95.8010 (10)º	*T* = 298 (2) K
*V* = 1220.6 (2) Å^3^	Flake, brown
*Z* = 4	0.23 × 0.20 × 0.15 mm

### Data collection

**Table:**

Bruker SMART CCD area-detector diffractometer	2156 independent reflections
Radiation source: fine-focus sealed tube	1581 reflections with *I* > 2σ(*I*)
Monochromator: graphite	*R*_int_ = 0.028
*T* = 298(2) K	θ_max_ = 25.0º
φ and ω scans	θ_min_ = 1.5º
Absorption correction: multi-scan(SADABS; Sheldrick, 1996)	*h* = −14→16
*T*_min_ = 0.940, *T*_max_ = 0.961	*k* = −14→9
6042 measured reflections	*l* = −8→8

### Refinement

**Table:**

Refinement on *F*^2^	Secondary atom site location: difference Fourier map
Least-squares matrix: full	Hydrogen site location: inferred from neighbouring sites
*R*[*F*^2^ > 2σ(*F*^2^)] = 0.045	H-atom parameters constrained
*wR*(*F*^2^) = 0.128	*w* = 1/[σ^2^(*F*_o_^2^) + (0.0528*P*)^2^ + 1.108*P*] where *P* = (*F*_o_^2^ + 2*F*_c_^2^)/3
*S* = 1.05	(Δ/σ)_max_ < 0.001
2156 reflections	Δρ_max_ = 0.44 e Å^−^^3^
165 parameters	Δρ_min_ = −0.44 e Å^−^^3^
Primary atom site location: structure-invariant direct methods	Extinction correction: none

### Special details

**Table:**

Geometry. All e.s.d.'s (except the e.s.d. in the dihedral angle between two l.s. planes) are estimated using the full covariance matrix. The cell e.s.d.'s are taken into account individually in the estimation of e.s.d.'s in distances, angles and torsion angles; correlations between e.s.d.'s in cell parameters are only used when they are defined by crystal symmetry. An approximate (isotropic) treatment of cell e.s.d.'s is used for estimating e.s.d.'s involving l.s. planes.
Refinement. Refinement of *F*^2^ against ALL reflections. The weighted *R*-factor *wR* and goodness of fit *S* are based on *F*^2^, conventional *R*-factors *R* are based on *F*, with *F* set to zero for negative *F*^2^. The threshold expression of *F*^2^ > σ(*F*^2^) is used only for calculating *R*-factors(gt) *etc*. and is not relevant to the choice of reflections for refinement. *R*-factors based on *F*^2^ are statistically about twice as large as those based on *F*, and *R*- factors based on ALL data will be even larger.

### Fractional atomic coordinates and isotropic or equivalent isotropic displacement parameters (Å^2^)

**Table:**

	*x*	*y*	*z*	*U*_iso_*/*U*_eq_	
N1	0.70555 (16)	0.62949 (19)	0.4602 (3)	0.0366 (6)	
O1	0.22901 (17)	0.5894 (2)	0.3632 (3)	0.0621 (7)	
H1	0.2437	0.5289	0.4103	0.093*	
O2	0.27564 (15)	0.5149 (2)	0.0717 (3)	0.0596 (7)	
O3	0.26181 (15)	0.71745 (19)	0.1207 (3)	0.0581 (7)	
O4	0.90263 (14)	0.64516 (16)	0.5898 (3)	0.0409 (5)	
S1	0.28904 (5)	0.60909 (6)	0.18999 (10)	0.0392 (2)	
C1	0.41271 (19)	0.6154 (2)	0.2690 (3)	0.0323 (6)	
C2	0.4477 (2)	0.7072 (2)	0.3712 (4)	0.0379 (7)	
H2	0.4062	0.7648	0.3957	0.045*	
C3	0.5434 (2)	0.7130 (2)	0.4362 (4)	0.0375 (7)	
H3	0.5661	0.7736	0.5073	0.045*	
C4	0.60639 (19)	0.6285 (2)	0.3960 (3)	0.0321 (6)	
C5	0.5707 (2)	0.5367 (2)	0.2959 (4)	0.0389 (7)	
H5	0.6120	0.4790	0.2711	0.047*	
C6	0.4741 (2)	0.5301 (2)	0.2326 (4)	0.0389 (7)	
H6	0.4508	0.4683	0.1656	0.047*	
C7	0.7498 (2)	0.7230 (2)	0.4686 (4)	0.0381 (7)	
H7	0.7155	0.7862	0.4270	0.046*	
C8	0.8478 (2)	0.7368 (2)	0.5371 (4)	0.0383 (7)	
C9	0.9011 (2)	0.8304 (3)	0.5670 (4)	0.0472 (8)	
H9	0.8809	0.9033	0.5424	0.057*	
C10	0.9932 (2)	0.7963 (3)	0.6424 (4)	0.0476 (8)	
H10	1.0452	0.8426	0.6781	0.057*	
C11	0.9918 (2)	0.6844 (3)	0.6528 (4)	0.0425 (7)	
C12	1.0641 (2)	0.5993 (3)	0.7175 (5)	0.0583 (9)	
H12A	1.1232	0.6355	0.7616	0.087*	
H12B	1.0750	0.5505	0.6179	0.087*	
H12C	1.0409	0.5568	0.8149	0.087*	

### Atomic displacement parameters (Å^2^)

**Table:**

	*U*^11^	*U*^22^	*U*^33^	*U*^12^	*U*^13^	*U*^23^
N1	0.0333 (13)	0.0386 (14)	0.0368 (13)	−0.0029 (10)	−0.0015 (10)	0.0024 (10)
O1	0.0497 (14)	0.0632 (16)	0.0740 (17)	−0.0002 (12)	0.0089 (13)	0.0110 (13)
O2	0.0425 (13)	0.0767 (17)	0.0577 (14)	−0.0077 (11)	−0.0044 (11)	−0.0265 (12)
O3	0.0416 (12)	0.0638 (15)	0.0671 (16)	0.0026 (11)	−0.0034 (11)	0.0290 (12)
O4	0.0364 (11)	0.0362 (11)	0.0490 (12)	−0.0018 (8)	−0.0017 (9)	0.0015 (9)
S1	0.0308 (4)	0.0463 (5)	0.0393 (4)	−0.0031 (3)	−0.0026 (3)	0.0019 (3)
C1	0.0306 (14)	0.0370 (15)	0.0288 (14)	−0.0035 (12)	0.0009 (11)	0.0023 (12)
C2	0.0352 (15)	0.0367 (16)	0.0418 (16)	0.0035 (12)	0.0041 (13)	−0.0049 (13)
C3	0.0384 (16)	0.0369 (16)	0.0362 (15)	−0.0046 (12)	−0.0003 (13)	−0.0077 (12)
C4	0.0321 (14)	0.0342 (15)	0.0293 (14)	−0.0023 (11)	−0.0008 (11)	0.0039 (11)
C5	0.0334 (15)	0.0353 (16)	0.0476 (17)	0.0026 (12)	0.0017 (13)	−0.0045 (13)
C6	0.0380 (16)	0.0345 (16)	0.0433 (17)	−0.0046 (12)	−0.0003 (13)	−0.0075 (13)
C7	0.0354 (15)	0.0371 (16)	0.0406 (16)	−0.0012 (13)	−0.0013 (13)	0.0033 (13)
C8	0.0375 (16)	0.0371 (16)	0.0394 (16)	−0.0013 (12)	−0.0012 (13)	0.0025 (13)
C9	0.0441 (18)	0.0349 (17)	0.060 (2)	−0.0035 (14)	−0.0054 (15)	0.0040 (15)
C10	0.0363 (17)	0.0469 (19)	0.057 (2)	−0.0104 (14)	−0.0080 (15)	−0.0009 (15)
C11	0.0327 (16)	0.0493 (19)	0.0443 (17)	−0.0026 (13)	−0.0020 (13)	0.0005 (14)
C12	0.0444 (19)	0.057 (2)	0.072 (2)	0.0109 (16)	−0.0017 (17)	0.0028 (18)

### Geometric parameters (Å, °)

**Table:**

N1—C7	1.278 (3)	C4—C5	1.386 (4)
N1—C4	1.418 (3)	C5—C6	1.385 (4)
O1—S1	1.608 (2)	C5—H5	0.9300
O1—H1	0.8200	C6—H6	0.9300
O2—S1	1.424 (2)	C7—C8	1.420 (4)
O3—S1	1.431 (2)	C7—H7	0.9300
O4—C11	1.368 (3)	C8—C9	1.352 (4)
O4—C8	1.372 (3)	C9—C10	1.409 (4)
S1—C1	1.768 (3)	C9—H9	0.9300
C1—C6	1.377 (4)	C10—C11	1.344 (4)
C1—C2	1.392 (4)	C10—H10	0.9300
C2—C3	1.375 (4)	C11—C12	1.478 (4)
C2—H2	0.9300	C12—H12A	0.9600
C3—C4	1.394 (4)	C12—H12B	0.9600
C3—H3	0.9300	C12—H12C	0.9600
			
C7—N1—C4	118.4 (2)	C1—C6—C5	119.9 (3)
S1—O1—H1	109.5	C1—C6—H6	120.0
C11—O4—C8	106.5 (2)	C5—C6—H6	120.0
O2—S1—O3	119.29 (15)	N1—C7—C8	124.3 (3)
O2—S1—O1	108.48 (14)	N1—C7—H7	117.8
O3—S1—O1	105.76 (13)	C8—C7—H7	117.8
O2—S1—C1	107.28 (13)	C9—C8—O4	109.6 (2)
O3—S1—C1	107.07 (13)	C9—C8—C7	130.5 (3)
O1—S1—C1	108.60 (13)	O4—C8—C7	119.9 (2)
C6—C1—C2	119.9 (3)	C8—C9—C10	106.9 (3)
C6—C1—S1	120.9 (2)	C8—C9—H9	126.6
C2—C1—S1	119.2 (2)	C10—C9—H9	126.6
C3—C2—C1	120.2 (3)	C11—C10—C9	107.1 (3)
C3—C2—H2	119.9	C11—C10—H10	126.5
C1—C2—H2	119.9	C9—C10—H10	126.5
C2—C3—C4	120.2 (3)	C10—C11—O4	110.0 (3)
C2—C3—H3	119.9	C10—C11—C12	133.9 (3)
C4—C3—H3	119.9	O4—C11—C12	116.1 (3)
C5—C4—C3	119.2 (3)	C11—C12—H12A	109.5
C5—C4—N1	118.0 (2)	C11—C12—H12B	109.5
C3—C4—N1	122.8 (2)	H12A—C12—H12B	109.5
C6—C5—C4	120.6 (3)	C11—C12—H12C	109.5
C6—C5—H5	119.7	H12A—C12—H12C	109.5
C4—C5—H5	119.7	H12B—C12—H12C	109.5
			
O2—S1—C1—C6	−8.0 (3)	C2—C1—C6—C5	−0.8 (4)
O3—S1—C1—C6	−137.2 (2)	S1—C1—C6—C5	−179.9 (2)
O1—S1—C1—C6	109.0 (2)	C4—C5—C6—C1	0.0 (4)
O2—S1—C1—C2	172.8 (2)	C4—N1—C7—C8	177.3 (3)
O3—S1—C1—C2	43.7 (3)	C11—O4—C8—C9	−0.2 (3)
O1—S1—C1—C2	−70.1 (2)	C11—O4—C8—C7	−178.6 (3)
C6—C1—C2—C3	−0.1 (4)	N1—C7—C8—C9	−174.2 (3)
S1—C1—C2—C3	179.1 (2)	N1—C7—C8—O4	3.8 (4)
C1—C2—C3—C4	1.7 (4)	O4—C8—C9—C10	−0.3 (4)
C2—C3—C4—C5	−2.4 (4)	C7—C8—C9—C10	177.9 (3)
C2—C3—C4—N1	−179.6 (2)	C8—C9—C10—C11	0.6 (4)
C7—N1—C4—C5	146.0 (3)	C9—C10—C11—O4	−0.8 (4)
C7—N1—C4—C3	−36.9 (4)	C9—C10—C11—C12	179.8 (4)
C3—C4—C5—C6	1.6 (4)	C8—O4—C11—C10	0.7 (3)
N1—C4—C5—C6	178.8 (2)	C8—O4—C11—C12	−179.8 (3)

### Hydrogen-bond geometry (Å, °)

**Table:**

*D*—H···*A*	*D*—H	H···*A*	*D*···*A*	*D*—H···*A*
O1—H1···N1^i^	0.82	2.21	3.025 (3)	176

Symmetry codes: (i) −*x*+1, −*y*+1, −*z*+1.
